# Outcomes for Younger Patients with Femoral Neck Fractures

**DOI:** 10.2106/JBJS.23.00582

**Published:** 2024-12-23

**Authors:** Boris C. Wagner, William M. Oliver, Katrina R. Bell, Chloe E.H. Scott, John F. Keating, Timothy O. White, Nick D. Clement, Andrew D. Duckworth

**Affiliations:** 1 Edinburgh Orthopaedics, Royal Infirmary of Edinburgh, Edinburgh, Scotland; 2 Centre for Population Health Sciences, Usher Institute, University of Edinburgh, Edinburgh, Scotland

## Abstract

**Background:**

There is a paucity of longer-term outcome data in younger adult patients who undergo fixation for an intracapsular hip fracture. The aims of this study were to evaluate the outcomes for young adult patients undergoing intracapsular hip fracture fixation and to assess factors associated with failure and patient-reported outcome measures (PROMs).

**Methods:**

From 2008 to 2018, 112 consecutive patients ≤60 years of age (mean age, 48 years [range, 20 to 60 years]; 54% male) were retrospectively identified as having undergone fixation of an intracapsular hip fracture. Displaced fractures (n = 81) were more common than nondisplaced or minimally displaced fractures (n = 31). Failure was defined as loss of fixation, nonunion, or osteonecrosis. PROMs that were assessed included the Oxford Hip Score (OHS), Forgotten Joint Score (FJS), EuroQol 5-Dimension (EQ-5D) index and Visual Analogue Scale (EQ-VAS), and University of California Los Angeles (UCLA) Activity Scale.

**Results:**

Eighty-six patients (77%) had union without evidence of failure, and 26 patients (23%) had a failure, including loss of fixation (6 patients; 5.4%), nonunion (5 patients; 4.5%), and osteonecrosis (16 patients; 14.3%). Overall, 39 patients (35%) required secondary surgery, with hardware removal (21 patients; 18.8%) and total hip arthroplasty (21 patients; 18.8%) being the most frequent procedures. Long-term functional outcomes were obtained for 81 patients (72%) at a mean of 7 years (range, 2.8 to 12.8 years). The median OHS was 47 (interquartile range [IQR], 40-48), the median FJS was 75 (IQR, 49-85), the median EQ-5D index was 1.00 (IQR, 0.77-1.00), and the median EQ-VAS was 90 (IQR, 70-95). The mean UCLA score fell from 6.8 preinjury to 6.0 postinjury (p < 0.001). Compared with the patients who had primary union, those who had a complication had significantly lower median OHS scores (44.5 versus 47, p = 0.008), EQ-5D index scores (0.825 versus 1.00, p = 0.001), EQ-VAS scores (70 versus 90, p = 0.01), and UCLA scores (4.5 versus 6.5, p = 0.001).

**Conclusions:**

One in 4 young adult patients undergoing intracapsular hip fracture fixation had a failure. Failure was associated with inferior long-term function and health-related quality of life.

**Level of Evidence:**

Therapeutic Level III. See Instructions for Authors for a complete description of levels of evidence.

Hip fractures are common injuries, with a worldwide annual incidence projected to increase to 2.6 million by 2025 and 4.5 million by 2050^1^. In older adults, 90% of hip fractures are caused by low-energy falls from standing height^2^. In contrast, hip fractures in younger adults, which account for only 3% of all hip fractures, are more commonly due to higher-energy mechanisms and often result in displaced fracture patterns^3^.

The treatment of displaced intracapsular hip fractures in elderly patients routinely involves arthroplasty, either hemiarthroplasty or total hip arthroplasty (THA)^4^. However, in younger patients with higher functional demands, joint preservation is the primary aim, with reduction and internal fixation typically being employed^5,6^. Due to the biomechanical properties of intracapsular fractures and the potential compromise to femoral head blood supply, the risks of failure include loss of fixation, nonunion, and osteonecrosis^7^. These risks are greatest within the first 2 years following injury^8^. The osteonecrosis rate has been reported to be as high as 80%^9^, and the overall rate of reoperation has been reported to be 30%^10^.

Medical comorbidities such as chronic respiratory disease, renal disease, and excessive alcohol use, along with poor fracture reduction, have been associated with fixation failure in younger adults with hip fractures^11,12^. The importance of other factors such as time to fixation, choice of implant, and the role of capsular decompression remain controversial^12-14^. Two recent systematic reviews showed that fewer than half of studies of hip fractures in younger adults included any functional or health-related quality of life (HRQoL) data^15,16^. Of these, only 1 assessed outcomes beyond 2 years^17^. A greater understanding of the longer-term outcomes of these injuries is of paramount importance to younger adults with hip fractures and the surgeons who treat them.

The aims of this study were to evaluate the outcomes of fixation of intracapsular hip fractures in younger adults (≤60 years of age), to assess any factors associated with the failure of surgical treatment, and to report patient-reported outcome measures (PROMs) in the longer term.

## Materials and Methods

Using an established trauma database, we retrospectively identified a consecutive series of adult patients ≤60 years of age who had undergone internal fixation for the treatment of an intracapsular hip fracture. Intertrochanteric or basicervical hip fracture patterns were not considered. On the basis of these inclusion criteria, 140 patients were identified over an 11-year period (January 2008 to December 2018). Patients with periprosthetic fractures (n = 1) or stress fractures (n = 7) were excluded. Patients with incomplete radiographic or clinical follow-up (less than a minimum of 1 year) were also excluded (n = 20). The remaining 112 patients formed the study cohort. The study was reviewed by our Research Ethics Service (reference NR/2005001).

### Patient and Injury Characteristics

Data regarding demographic characteristics, medical comorbidities, alcohol and smoking status, mechanism of injury, and preinjury mobility were collected retrospectively from the electronic patient record (EPR). Socioeconomic deprivation was determined using the Scottish Index of Multiple Deprivation (SIMD)^18^. Excessive alcohol use was defined as exceeding the current national guidance of 14 units per week^19^. Preinjury mobility was defined as mobilizing either independently or with walking aids.

The mechanism of injury was categorized as either low-energy (fall from standing height) or high-energy (fall from greater height, road traffic accident, or sporting injury). Fractures were classified by 2 independent observers (W.M.O. and K.R.B.) on the basis of preoperative anteroposterior and lateral radiographs with use of the Garden system^20^, with disagreements resolved through consensus with a third senior author (A.D.D.).

### Management

Time to fixation was the interval between presentation to the emergency department and the first fluoroscopic image. Time to fixation was further classified as ≤24 hours or >24 hours^12,21^. Time to fixation could not be determined for 4 patients (3.6%), and another 4 patients (3.6%) underwent preoperative computed tomographic (CT) scans to confirm the diagnosis. The median time to fixation was 16.1 hours (range, 0.5 to 73.6 hours; interquartile range [IQR] width, 14.2 hours). A delay in time to fixation of >24 hours occurred in 29 patients (28% of 104) due a delay in diagnosis (n = 3), being medically unfit (n = 6), or an unspecified reason (n = 20).

**Fig. 1:**
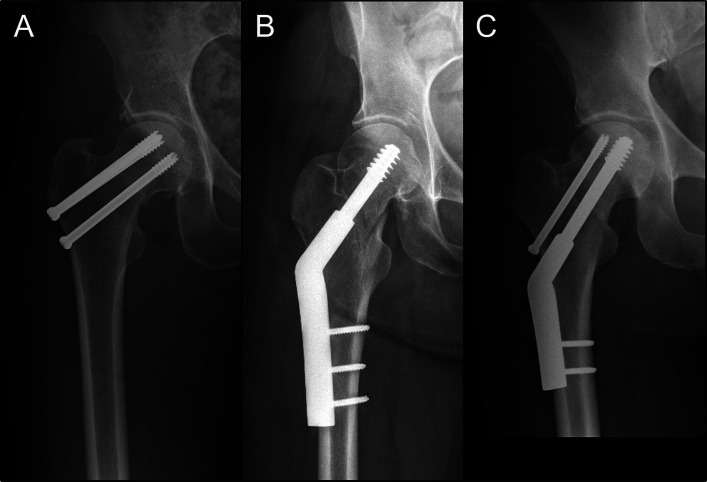
Anteroposterior radiographs showing the internal fixation implant options utilized in the present study, including cannulated screws (**Fig. 1-A**), a dynamic hip screw (**Fig. 1-B**), and a dynamic hip screw plus an antirotation screw (**Fig. 1-C**).

Complete operative notes were available for 97 of the 112 patients. The operations were performed by a supervised senior trainee/fellow (85%; 82 of 97) or a consultant orthopaedic trauma surgeon (15%; 15 of 97). Ninety-two patients (95%) underwent closed reduction, and 5 patients (5%) underwent open reduction (all via an anterior Smith-Petersen approach). Of the 92 patients with closed reduction, 6 (7%) underwent capsular decompression with hematoma aspiration. Definitive fixation was performed via a direct lateral approach in all cases. All patients underwent internal fixation (Fig. 1), with partially threaded cannulated screws (58%; 65 of 112), dynamic hip screws (14.3%; 16 of 112), or dynamic hip screws plus antirotation (cannulated) screws (27.7%; 31 of 112). Fixation with cannulated screws involved the use of 3 screws in 1 of the following configurations: inverted triangle (49%; 32 of 65), parallel vertical (28%; 18 of 65), and triangle (23%; 15 of 65). Fracture reduction or malreduction was determined on postoperative anteroposterior and lateral radiographs with use of the Garden Alignment Index, with 160° to 180° deemed satisfactory^22^.

### Short-Term Follow-up

Short-term outcomes were determined from the EPR in conjunction with a review of radiographic images. Adequate follow-up was defined as documented radiographic and/or clinical assessment at ≥12 months following surgery. Routine policy at our institution is to review patients at 6 weeks and at 3, 9, 12, 18, and 24 months postoperatively. Union was defined as hip pain improvement/resolution with radiographic evidence of fracture bridging and reestablishment of the trabecular pattern on anteroposterior and lateral radiographs^23^. Failure was defined as loss of fixation (fracture collapse of >10 mm and/or screw cut-out)^24^, nonunion (pain and radiographic evidence of nonunion at 6 months postoperatively), or osteonecrosis (radiographic evidence of subchondral sclerosis and/or segmental collapse). These definitions were based on a previous study and are consistent with existing literature^12^. Infection and the requirement and indication for revision surgery were recorded.

### Long-Term Follow-up

PROMs were collected with use of a telephone questionnaire at a minimum of 2 years postoperatively. The outcome measures included the Oxford Hip Score (OHS)^25^, Forgotten Joint Score (FJS)^26^, EuroQol 5-Dimension (EQ-5D) 3-Level health index^27^ and Visual Analogue Scale (EQ-VAS) score, and University of California Los Angeles (UCLA) Activity Scale^28^. Published mean population reference norms were available for the OHS, EQ-5D, and EQ-VAS^29,30^. The OHS, EQ-5D index, and EQ-VAS for age-matched individuals in the normal population were determined for each patient.

### Statistical Analysis

Parametric continuous data were analyzed with use of the unpaired Student t test. Nonparametric continuous data were analyzed with use of the Mann-Whitney U test or the Wilcoxon signed-rank test for paired measures. Dichotomous data were examined with bivariate analysis with use of either the Fisher exact test (n ≤ 5) or the Pearson chi-square test (n > 5) to determine factors associated with failure. The level of significance was set at p < 0.05.

## Results

**Fig. 2:**
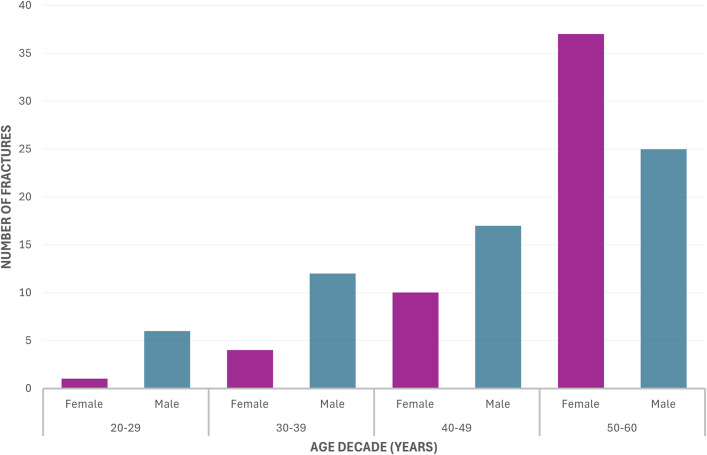
Bar graph showing the number of fractures by sex and age.

The patients included 60 men (53.6%) and 52 women (46.4%), and the mean age (and standard deviation) was 48 ± 9.8 years (range, 20 to 60 years) (Table I). The mean age of the female patients was greater than that of the male patients (51 versus 45 years; p = 0.002). Most fractures (79.5%; 89 of 112) occurred in patients ≥40 years of age (Fig. 2). The most common mechanism of injury was a fall from standing height (49.1%; 55 of 112), particularly among women (female:male ratio, 2.2:1.0). High-energy mechanisms, including falls from height (15.2%; 17 of 112), motor vehicle accidents (14.3%, 16 of 112), and sporting injuries (21.4%; 24 of 112), were more common in men (male:female ratio, 1.1:1.0, 4:1, and 7:1, respectively). Displaced fractures (Garden types III and IV; n = 81) were common (Table I).

**TABLE I:** Demographic Characteristics and Fracture Classification (N = 112)

Age* *(yr)*	48 ± 9.8 (20 to 60)
Sex *(no. of patients)*	
Male	60 (53.6%)
Female	52 (46.4%)
BMI* *(kg/m*^*2*^*)*	24.1 ± 3.9 (16.7 to 34.5)
Preinjury mobility *(no. of patients)*	
Unassisted	107 (95.5%)
With aids	5 (4.5%)
Side of injury *(no. of patients)*	
Left	59 (52.7%)
Right	53 (47.3%)
Garden classification *(no. of patients)*	
I	19 (17.0%)
II	12 (10.7%)
III	40 (35.7%)
IV	41 (36.6%)

* The values are given as the mean and the standard deviation, with the range in parentheses. BMI = body mass index.

### Short-Term Outcomes

**Fig. 3:**
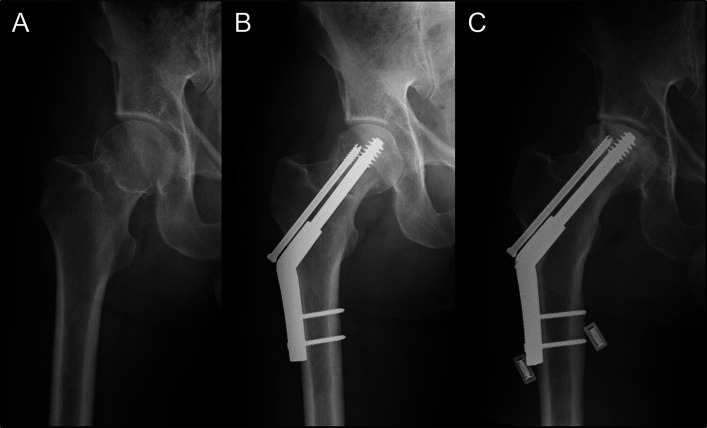
Anteroposterior radiographs of the hip in a 47-year-old man with an intracapsular femoral neck fracture, made immediately following the injury (**Fig. 3-A**), 6 weeks postoperatively (**Fig. 3-B**), and 2 years postoperatively, by which time femoral head osteonecrosis had developed (**Fig. 3-C**).

Radiographs were reviewed at a mean of 3.0 years (range, 1.0 to 9.0 years). Union without evidence of failure at any point was achieved in 86 patients (77%). The remaining 26 patients (23%) had a failure of surgical management. Loss of fixation occurred in 6 patients (5.4%) because of fracture collapse of >10 mm (n = 3) and screw cut-out (n = 3). Nonunion occurred in 5 patients (4.5%), and osteonecrosis occurred in 16 patients (14.3%) (Fig. 3). One patient had both loss of fixation (at 1 month) and subsequent osteonecrosis (at 14 months). Three patients (2.7%) had a postoperative infection; of those 3 patients, 1 had an open reduction. One patient (0.9%) had a superficial wound infection that resolved after treatment with oral antibiotics, and 2 patients (1.8%) had a deep infection requiring surgical irrigation and debridement. Two patients (1.8%) had a deep vein thrombosis, and both were managed with therapeutic anticoagulation. Excessive alcohol use was more frequently reported in the failure group (p = 0.04) (Table II). Time to fixation of >24 hours (p = 0.017) and fracture malreduction (p = 0.002) were also more common in the failure group (Table II). All malreductions were in varus on the anteroposterior radiograph (Table II).

**TABLE II:** Demographic, Injury, and Treatment Factors and Their Association with Failure*

	Union	Failure	P Value
Total *(no. of patients)*	86 (76.8%) of 112	26 (23.2%) of 112	NA
Men:women *(no. of patients)* [M:F ratio]	46:40 [1.2:1.0]	14:12 [1.2:1.0]	0.97†
Age# *(yr)*	48 (20 to 60)	48.7 (25 to 60)	0.74‡
BMI# *(kg/m*^*2*^*)*	23.9 (16.7 to 34.5)	24.7 (16.9 to 34.1)	0.42‡
Smoking *(no. of patients)*	22 (27.2%) of 81	11 (45.8%) of 24	0.08†
Excessive alcohol use *(no. of patients)*	7 (8.5%) of 82	6 (24%) of 25	**0.04** †
Preexisting mobility problems *(no. of patients)*	4 (4.7%)	1 (3.8%)	0.67§
Comorbidities *(no. of patients)*			
Hypertension	6 (7%)	4 (15.4%)	0.237§
Diabetes	3 (3.5%)	0 (0%)	1.00§
Cardiovascular disease	4 (4.7%)	0 (0%)	0.572§
Chronic respiratory disease	9 (10.5%)	4 (15.4%)	0.495§
Chronic liver disease	4 (4.7%)	1 (3.8%)	1.00§
Chronic kidney disease	1 (1.2%)	0 (0%)	1.00§
Cerebrovascular disease	2 (2.3%)	2 (7.7%)	0.230§
Epilepsy	2 (2.3%)	0 (0%)	1.00§
Multiple sclerosis	3 (3.5%)	0 (0%)	1.00§
Cancer	4 (4.7%)	1 (3.8%)	1.00§
Depression	7 (8.1%)	4 (15.4%)	0.277§
Eating disorder	1 (1.2%)	0 (0%)	1.00§
Drugs *(no. of patients)*			
Steroids	3 (3.5%)	1 (3.8%)	1.00§
Proton pump inhibitor	7 (8.1%)	2 (7.7%)	1.00§
Selective serotonin reuptake inhibitor	4 (4.7%)	1 (3.8%)	1.00§
Anti-epileptics	4 (4.7%)	1 (3.8%)	1.00§
SIMD quintiles *(no. of patients)*			0.538†
1 (most deprived)	6 (7%)	4 (15.4%)	
2	16 (18.6%)	6 (23.1%)	
3	23 (26.7%)	6 (23.1%)	
4	9 (10.5%)	3 (11.5%)	
5 (least deprived)	32 (37.2%)	6 (23.1%)	
Missing	0	1	
Fracture type *(no. of patients)*			0.272†
Nondisplaced (Garden I/II)	26 (30.2%)	5 (19.2%)	
Displaced (Garden III/IV)	60 (69.8%)	21 (80.8%)	
Time to fixation >24 hr *(no. of patients)*	18 (21.4%) of 84	11 (45.8%) of 24	**0.017** †
Open reduction *(no. of patients)*	3 (4.0%) of 75	2 (9.1%) of 22	0.317§
Capsular decompression/hematoma aspiration *(no. of patients)*	4 (5.3%) of 75	2 (9.1%) of 22	0.616§
Quality of reduction from postreduction radiograph *(no. of patients)*			
Anteroposterior malreduction	4 (4.7%)	7 (26.9%)	**0.002** §
Lateral malreduction	0 (0%)	1 (3.8%)	0.225§
Anteroposterior or lateral malreduction	4 (4.7%)	7 (26.9%)	**0.002** §
Anteroposterior and lateral malreduction	0 (0%)	1 (3.8%)	0.225§
Choice of implant *(no. of patients)*			0.434†
Cannulated screws	53 (61.6%)	12 (46.2%)	
Dynamic hip screw	11 (12.8%)	5 (19.2%)	
Dynamic hip screw + antirotation screw	22 (25.6%)	9 (34.6%)	

* Bold p values indicate significance. NA = not applicable, BMI = body mass index, SIMD = Scottish Index of Multiple Deprivation.

† Chi-square test.

‡ Unpaired t test.

# The values are given as the mean, with the range in parentheses.

§ Fisher exact test.

#### Additional Procedures

Overall, 39 patients (35%) underwent an additional procedure. Two patients (1.8%) underwent resection arthroplasty because of deep infection. One patient underwent revision fixation with a valgus osteotomy and 95° blade-plate because of screw cut-out and progressive varus deformity at 8 weeks postoperatively. Fifteen patients underwent revision to THA because of loss of fixation (1 patient; 0.9%), nonunion (4 patients; 3.6%), and osteonecrosis (10 patients; 8.9%). Two additional patients were scheduled for THA but did not proceed due to medical contraindications. Twenty-one patients (18.8%) underwent removal of hardware; of those, 6 patients subsequently underwent revision to THA because of pain (1 patient; 0.9%), posttraumatic osteoarthritis (1 patient; 0.9%), and osteonecrosis (4 patients; 3.6%). The overall salvage THA rate was 18.8% (21 of 112), or 20.5% (23 of 112) by intention-to-treat.

### Long-Term Outcomes

PROMs were obtained for 81 patients (72%) at a mean of 6.9 years (range, 2.8 to 12.8 years). The mean age of these patients was 56 years (range, 25 to 71 years). Of the remaining 31 patients, 8 (7%) had died, 19 were uncontactable, 2 declined, and 2 had incapacity. The median OHS was 47 (IQR, 40 to 48), which did not differ significantly from the age-matched population reference of 45.7^29^ (p = 0.692). For 18 (22%) of the 81 patients, the OHS was >1 minimal clinically important difference (MCID) below the value for age-matched individuals in the reference population^31^. The median EQ-5D was significantly higher (p = 0.002) than the age-matched population reference, with the score for 16 (20%) of the 81 patients being >1 MCID below the reference^32^ (Table III). The median EQ-VAS was higher than the age-matched population mean^30^, although this difference was not significant (p = 0.528), and 24 (30%) of the 81 patients had scores that were >1 MCID below the value for the age-matched population^32^. The median FJS was 75 (IQR, 49 to 85). The mean UCLA Activity Scale decreased from 6.8 to 6.0 (p < 0.001).

**TABLE III:** Comparison of PROMs at Time of Long-Term Follow-up to Reference Population Values*

PROM	Age-Matched Normal Population	Study Group†	P Value‡
OHS	45.7	47 (8)	0.692§
FJS	NA	75 (36.5)	NA
EQ-5D Index	0.804	1.00 (0.24)	**0.002** §
EQ-VAS	81.7	90 (25)	0.528§
Pain VAS	NA	90 (30)	NA

* NA = not applicable.

† The values are given as the median at the time of follow-up, with the IQR width in parentheses.

‡ Bold values indicate significance.

§ Single-sample Wilcoxon signed-rank test.

Compared with primary union, failure was associated with an inferior OHS (44.5 versus 47; p = 0.008), EQ-5D index (0.825 versus 1.00; p = 0.001), EQ-VAS (70 versus 90; p = 0.01), VAS for pain (55 versus 90; p < 0.001), and UCLA Activity Scale (4.5 versus 6.5; p = 0.001). However, patients with a failure who did not undergo salvage THA reported much lower PROMs (Table IV).

**TABLE IV:** Comparison of PROMs Based on Outcome and Salvage Operation in Patients with Long-Term Follow-up

PROM	Entire Cohort (N = 81)	Union (N = 61)	Failure	
			Salvage Total Hip Arthroplasty (N = 15)	No Salvage Total Hip Arthroplasty (N = 5)
OHS*	47 (8)	47 (4)	46 (13.5)	12 (14)
FJS*	75 (36.5)	75 (33.4)	68.8 (40.6)	19.2 (14)
EQ-5D Index*	1.00 (0.24)	1.00 (0)	0.85 (0.23)	0.08 (0.59)
EQ-VAS*	90 (25)	90 (15)	85 (32.5)	50 (20)
Pain VAS*	90 (30)	90 (20)	70 (47.5)	20 (20)
UCLA Activity Scale (postinjury)†	5.98 ± 2.3	6.5 ± 2.1	5.3 ± 1.8	2 ± 1.7

* The values are given as the median, with the interquartile range in parentheses.

† The values are given as the mean and the standard deviation.

## Discussion

This large study of younger adult patients who underwent fixation of an intracapsular hip fracture demonstrated that the majority of patients (77%) progressed to union without failure following primary surgery and had equivalent hip-specific function and HRQoL compared with an age-matched population. However, almost 1 in 4 patients had a failure of primary fixation, with osteonecrosis being the most frequent reason (14.3% of patients). At an average of 7 years after the injury, HRQoL and joint-specific PROMs were inferior among patients who had a failure of fixation compared with those who had union without failure. Identifying risk factors associated with failure may confer longer-term benefits for younger adult patients with hip fractures.

The rates of union without complication (77%) and of failure (23%) are comparable with existing data^3,12,33-35^. A systematic review estimated a pooled rate of osteonecrosis of 14.3%^7^, which is consistent with the findings of the present study. Although the reported osteonecrosis rate has varied widely (from 0% to 80%)^9,10,12,36^, these differences may be explained by the age ranges included, the exclusion of high-energy injury mechanisms in some studies, and the limited follow-up duration in much of the literature.

Alcohol has previously been associated with increased rates of complications following hip fractures in younger adults^12,37^. The explanation for this finding is likely multifactorial, as excessive alcohol use can lead to reduced bone quality^38,39^ as well as poorer compliance with postoperative weight-bearing restrictions^39,40^. Some studies have shown that early surgery (within 8 to 12 hours) reduces the risk of osteonecrosis^41^, and other studies have shown that surgery that is delayed by >20 or >24 hours is associated with higher complication rates^12,21^. Thus, the literature would suggest the importance of timely surgical intervention for hip fractures in younger adults. The importance of adequate reduction has been established, with an increased complication rate following malreduction^35,42^. With all postoperative radiographs available, the present study highlights the importance of achieving adequate intraoperative fracture reduction and avoidance of varus malreduction. Other important concepts to consider are fracture displacement and an understanding of the difficulties associated with assessing rotational reduction. Of note, only 6 patients who were treated with closed reduction underwent capsular decompression, of whom 2 had a failure. While there is debate regarding the value of capsular decompression in the context of closed reduction, there is minimal clinical evidence to suggest an impact on outcome^11^, and capsular decompression is not routinely performed in our center.

Analysis of the OHS and FJS indicated that, even in some cases in which initial management was successful, patients remained symptomatic^43^. Moreover, patients who had a failure of fixation had inferior PROMs and HRQoL in comparison with those who had union. Although salvage THA did appear to confer a partial improvement, the need for and consequences of subsequent surgery in this young population are notable. Overall, patients were found to have reduced activity levels, and, although a degree of age-related decline might be anticipated, there are no published data available for comparison.

Secondary THA is often indicated for the treatment of complications such as osteonecrosis or loss of fixation^11,44^, although secondary THA is associated with higher complication rates compared with primary THA for trauma^45,46^. Improved long-term implant survival potentially increases the viability of arthroplasty as a primary treatment for some younger patients with displaced fractures^47^ and may be considered for younger patients who are high-risk due to background factors associated with failure^12,48^. However, in patients with a history of excessive alcohol use or other factors associated with an increased risk of dislocation, a hemiarthroplasty or similar procedure may be preferable.

In the current study of young adults with hip fractures, men had a significantly lower mean age than women, and high-energy mechanisms have more frequently been seen in these younger patients^3^. Most fractures were caused by low-energy trauma, which was more commonly seen in older female patients^3,12,48^. This suggests 2 distinct types of hip fractures in adults ≤60 years of age: high-energy traumatic fractures in younger individuals, and low-energy early fragility fractures in patients with advanced biological age, in whom the risks of fixation failure may be higher^16^. This concept is consistent with the 7% mortality rate reported at a mean of just under 7 years.

The present study had several limitations associated with its retrospective design and relatively small sample size, including issues with clinical variation and truncation over the study period. There are also the issues of selection bias in relation to time to surgery, defining failure, and when or whether further surgery was undertaken. Attempts were made to account for these issues and confounders using regression analysis to find associations with failure, although this was not possible given the relatively small sample size and failure event rate. There was also the issue of surveillance bias, with 28% of patients being lost to long-term follow-up. However, the rate of failure in that group (19%; 6 of 31) was comparable with the rate of failure in the group with long-term outcomes (25%; 20 of 81). This finding suggests a limited impact of loss to follow-up on the overall findings of the study, although individual associations might have changed. Furthermore, consistent with all single-center studies, the generalizability of these data is unclear, as the findings are reflective of our experience and could be impacted by expertise bias.

However, the present study is one of the first to combine complications with longer-term functional and HRQoL measures. Although some studies have evaluated the outcomes in hip fractures in younger adults, the follow-up of almost 7 years in the present study represents the longest-term assessment of PROMs in the literature. Although baseline activity levels were obtained retrospectively (introducing recall bias), the data should still reflect a change in activity. A recent review^16^ suggested the importance of PROM data in the evaluation of hip fractures in younger adults, and, although we collected a wider variety of hip-specific and generic outcome measures, we were unable to assess some clinical outcomes, such as range of motion. Yet, given the breadth of PROMs that we used, we expect that these measures would detect any notable functional limitations.
